# Association between maternal exercise during pregnancy and attention-deficit/hyperactivity disorder among preschool children in Southwest China

**DOI:** 10.3389/fpubh.2024.1493580

**Published:** 2024-11-27

**Authors:** Yingying Liu, Hui Jiang, Zizheng Nie, Bin Yu, Xinyi Qiu, Hui Zuo, Shufen Han

**Affiliations:** ^1^School of Public Health, Hangzhou Normal University, Hangzhou, Zhejiang, China; ^2^School of Public Health, Soochow University Medical College, Suzhou, Jiangsu, China; ^3^Sichuan Bingzhe Technology Co., Ltd, Chengdu, Sichuan, China; ^4^Jiangsu Key Laboratory of Preventive and Translational Medicine for Geriatric Diseases & MOE Key Laboratory of Geriatric Diseases and Immunology, Suzhou Medical College of Soochow University, Suzhou, Jiangsu, China

**Keywords:** maternal exercise, pregnancy, preschool children, ADHD, mental health

## Abstract

**Objective:**

Maternal moderate-intensity exercise during pregnancy has important health benefits for the offspring, however, less is known about its association with offspring attention-deficit/hyperactivity disorder (ADHD). This study aimed to explore the association between maternal exercise during pregnancy and ADHD among preschool children in Southwest China.

**Methods:**

A web-based cross-sectional study was performed in 2021, and the revised Conners Parental Symptom Questionnaire and maternal exercise during pregnancy were obtained through a self-reported structured questionnaire. A binary logistic regression model was used to assess the association between maternal exercise during pregnancy and the likelihood of childhood ADHD after adjustment for covariates.

**Results:**

A total of 4,184 preschool children aged 3–6 years were included in our final analysis. Children whose mothers exercised for <20 min per day were more likely to be at risk of ADHD (6.3%), compared to those whose mothers exercised for more than 40 min (3.1%) or 20–40 min (2.8%) per day. Daily exercise of <20 min during pregnancy was associated with higher odds of childhood ADHD (adjusted OR = 2.11; 95% CI: 1.41, 3.16) after multivariable adjustment. The association of maternal exercise during pregnancy with childhood ADHD was similar in subgroups stratified by child's sex, and by maternal smoking, sleep duration and gestational anemia during pregnancy.

**Conclusion:**

Our findings highlight the importance of maternal moderate-intensity exercise during pregnancy for the prevention of childhood ADHD. Prospective studies are needed to confirm our findings in the future.

## 1 Introduction

Attention-deficit/hyperactivity disorder (ADHD) characterized by chronic symptoms of inattention, hyperactivity and impulsivity, is one of the most prevalent neurodevelopmental disorders in childhood (1). It is estimated that the global prevalence of ADHD in children under the age of 18 is 7.2% (2); in China, the prevalence is ~6.3% (3). Children diagnosed with ADHD are more likely to experience a variety of negative social problems, including poor interpersonal relationships, weak self-esteem, decreased quality of life, and an increased likelihood of substance use and related disorders (4, 5). ADHD frequently coexists with other childhood-onset neurodevelopmental and psychiatric disorders, resulting in substantial comorbidities (6). Approximately 60%–75% of individuals with ADHD have at least one additional mental disorder, potentially worsening the prognosis of ADHD (7).

Like most complex neuropsychiatric disorders, ADHD is characterized by multifactorial causation, with a highly heritable component (8). However, non-genetic factors also play a crucial role, especially during the prenatal and early postnatal periods (9). Epidemiological studies have demonstrated that exposure to a variety of prenatal and perinatal factors, such as maternal pre-pregnancy obesity or overweight, prenatal smoking, alcohol consumption, and drug use, significantly contributes to the development of childhood ADHD (10–14). Preventive measures that target non-genetic factors and optimize the intrauterine environment, may be crucial in reducing the risk of childhood ADHD.

Physical exercise is a vital element of overall health and wellbeing, contributing to the prevention and treatment of several diseases. Regular maternal exercise during pregnancy can help preventing pregnancy related disorders (15), including reducing the risk of prenatal and postnatal anxiety and depression (16). Also, prenatal exercise is safe and beneficial for the development of fetus (17). Recent studies demonstrated that maternal exercise during pregnancy was associated with reduced fetal adiposity, a decreased risk of preterm labor, and improved neurodevelopmental outcomes in offspring (18). Maternal exercise benefits the health of offspring through improving the intrauterine environment (19, 20). The intrauterine environment plays a crucial role in influencing the development of childhood ADHD (10, 11, 13). However, the precise influence of maternal exercise on the risk of ADHD in offspring remains uncertain. Therefore, the aim of the present study was to investigate the association between maternal exercise during pregnancy and childhood ADHD among preschool children through a cross-sectional study conducted in Southwest China.

## 2 Methods

### 2.1 Study design and participants

We performed a population-based cross-sectional study and collected information about preschool children along with their mothers during pregnancy between October and December 2021 in Chengdu, Southwest China. In our study, we used a multistage cluster sampling method to randomly select 30 kindergartens from a pool of ~2,100 kindergartens in Chengdu city. Subsequently, we randomly selected one class from the large, medium, or small class in each kindergarten, and all children in the chosen classes were included as participants in the study. A pretested self-administered questionnaire was distributed through the WJX platform (http://www.wjx.cn) and filled out by the children's parents or guardians. Prior to the administration of the survey, the study purpose and content were explained to the kindergarten directors. The head teacher of each selected class received uniform training to ensure the quality of the investigation. Then, the head teacher arranged an orientation meeting for the children's parents or guardians to explain the survey content and highlight essential precautions when filling out the questionnaire. Professional assistance for completing the questionnaire was also obtained through online or by telephone. One of the child's parents or guardians was invited to complete a survey regarding the basic information of children, as well as the Conners Parental Symptom Questionnaire (CPSQ) scale based on direct observations of the children at home over the past 6 months. Participants' mothers recalled and filled out relevant information about their pregnancies. To obtain more accurate data, we reviewed maternal health check-up records to access the mothers' health information during pregnancy through contacting community health institutions as much as possible.

A total of 4,360 preschool children aged 3–6 years were invited to participate in this study, and 4,326 questionnaires were collected. After eliminating questionnaires for children under the age of 3, as well as those with incomplete, unreliable, or incorrect information, and a response time of < 5 min, the final sample of this study consisted of 4,184 preschool children, including 2,000 girls and 2,184 boys. Informed consent was signed from all participants' parents or guardians before questionnaire administration. The study protocol was approved by the Institution Review Board of Soochow University (No. SUDA20210820H01).

### 2.2 Assessment of maternal exercise during pregnancy

According to the 2022 Dietary Guidelines for Chinese Residents (21), it is recommended that healthy pregnant women engage in regular physical activity for 20–40 min per day to promote the wellbeing of both themselves and their offspring. When completing the questionnaire about maternal exercise during pregnancy, the investigators assisted the participants' mothers in recalling their daily exercise duration and reviewed their pregnancy health records. Data on maternal exercise during pregnancy was collected by answering the following question: “How many minutes did you allocate daily to activities such as jogging, yoga, and other forms of physical activity during your pregnancy?” Responses were categorized as: < 20, 20–40, and ≥40 min per day. Maternal exercise of < 20 min per day during pregnancy was considered insufficient (21).

### 2.3 Definition of ADHD

Symptoms associated with ADHD in children were evaluated using the 1978 version of the revised and expanded CPSQ scale, which has been widely used in many countries to assess the total psycho-behavioral problems in children and adolescents aged 3–17 years (22). By analyzing the responses provided by one of the child's parents or guardians on the scale, the investigators determined whether these preschool children were at risk of ADHD. The CPSQ scale showed high reliability and validity and the Cronbach's alpha value of this present study was 0.933 (23).

The revised CPSQ included a 48-item screening test designed to assess conduct problems, learning problems, physical and mental disorders, hyperactivity-impulsivity, anxiety, or hyperactivity index. Among these, the hyperactivity index as a specific screening tool for children with symptoms of ADHD, included 10 behavioral statements with each item ranging from 0 (not at all) to 3 score (very often). Parents or guardians were asked to rate their children's behavior at home over the past 6 months. The cut-off value for a screening diagnosis of ADHD was determined by an average score on the hyperactivity index of 10 behavioral items, where a score of 1.5 or higher indicated the presence of symptoms (24).

### 2.4 Covariates

Based on literature reports on potential factors influencing childhood ADHD (25, 26) and the objective of our study, the following covariates were selected for inclusion in the present study. Basic information about the participants, including their sex and date of birth, was collected using a standardized self-administered questionnaire. Additionally, basic characteristics of the participating mothers, including maternal education level, pre-pregnancy body weight and height, smoking and alcohol drinking during pregnancy, sleep duration, morning sickness, folic acid supplementation, and prenatal education during pregnancy, and medical history (e.g. diabetes mellitus before pregnancy, gestational hyperglycemia including diabetes mellitus, pregnancy-induced hypertension, gestational anemia, anxiety or depression during pregnancy, and postpartum anxiety or depression), were self-reported by the mothers themselves.

Maternal education levels were classified into the following categories: junior high school and below, senior high school or secondary specialized school, junior college, and graduate and above. Body Mass Index (BMI) was calculated by dividing body weight by the square of body height (kg/m^2^). In alignment with the weight status classification for Chinese adults (27), BMI categories were defined as follows: BMI ≤ 18.5 kg/m^2^ for underweight, 18.5–23.9 kg/m^2^ for normal weight, 24–27.9 kg/m^2^ for overweight, and ≥28 kg/m^2^ for obesity. Alcohol drinking, morning sickness, and folic acid supplementation during pregnancy, and medical history were recorded as either yes or no. Smoking during pregnancy was documented as 0, 1–2, or ≥3 days per week, while prenatal education frequency was categorized as 0 times, 1–2 times, or ≥3 times per week. Sleep duration during pregnancy was classified as < 7, 7–8, or ≥8 h per day.

### 2.5 Data analysis

All characteristics of the data were described as numbers and percentages (%) for categorical variables, and the chi-squared test or Fisher's exact test was used to compare differences in child sociodemographic characteristics, as well as maternal lifestyle and health-related factors during pregnancy between the participants with or without ADHD. To examine the association between maternal exercise during pregnancy and childhood ADHD, we constructed three multivariable models in addition to the crude model: (1) Model 1 was adjusted for sex and age of the children; (2) Model 2 was adjusted based on Model 1, incorporating additional adjustments for maternal education level, pre-pregnancy BMI, smoking, alcohol drinking, prenatal education, sleep duration, morning sickness, and folic acid supplementation during pregnancy; (3) Model 3 was built on Model 2, with further adjustments for medical history factors including diabetes mellitus before pregnancy, gestational hyperglycemia, gestational hypertension, gestational anemia, anxiety or depression during pregnancy, and postpartum anxiety or depression. Additionally, stratified analyses were conducted by the child's sex, as well as by maternal smoking, sleep duration, and gestational anemia during pregnancy. Statistical analyses were performed using SPSS software (Version 23.0; IBM Corp., Armonk, NY, USA) and R software (Version 4.3.2; R Development Core Team, Vienna, Austria). A two-sided *p-*value of < 0.05 was considered as statistically significant.

## 3 Results

Of the 4,184 participants, 154 preschool children were at risk of ADHD according to the CPSQ assessment, with a higher prevalence in boys compared to girls (4.9% vs. 2.4%, *p* < 0.001). The mean age of the children was 4.3 ± 0.9 years. The percentage of participants' mothers who exercised during pregnancy for < 20, 20–40, and ≥40 min per day was 22.1%, 42.2%, and 35.7%, respectively. Among 154 children exhibiting symptoms of ADHD, 37.6% of their mothers reported exercising for < 20 min per day during pregnancy; in contrast, only 21.5% of mothers in the non-ADHD group reported similar levels of daily exercise. There were significant differences for the prevalence of ADHD among different categories of maternal exercise during pregnancy (*p* < 0.001). Additionally, preschool children whose mothers smoked during pregnancy, had shorter sleep duration, or were diagnosed with gestational anemia (all *p* < 0.05), exhibited the higher odds of developing ADHD. The detailed characteristics of participants and their mothers, stratified by childhood ADHD status, are showed in Table 1.

**Table 1:** Basic characteristics of the participating children and their mothers by the ADHD status.

**Characteristic**	**Overall (*n* = 4,184)**	**Without ADHD (*n* = 4,030)**	**With ADHD (*n* = 154)**	***p*-value**
**Sex**				< 0.001
Girls	2,000 (47.8)	1,952 (48.4)	48 (31.2)	
Boys	2,184 (52.2)	2,078 (51.6)	106 (68.8)	
**Age (years)**				0.221
3	996 (23.8)	958 (23.8)	38 (24.7)	
4	1,397 (33.4)	1,352 (33.5)	45 (29.2)	
5	1,417 (33.9)	1,355 (33.6)	62 (40.3)	
6	374 (8.9)	365 (9.1)	9 (5.8)	
**Maternal education level**				0.125
Junior high school and below	576 (13.8)	546 (13.5)	30 (19.5)	
Senior high school or secondary specialized school	1,395 (33.3)	1,341 (33.3)	54 (35.0)	
Junior college	1,214 (29.0)	1,174 (29.1)	40 (26.0)	
Graduate and above	999 (23.9)	969 (24.1)	30 (19.5)	
**Pre-pregnancy BMI (kg/m** ^2^ **)**				0.875
≤ 18.5	899 (21.5)	862 (21.4)	37 (24.0)	
18.5–23.9	2,888 (69.0)	2,785 (69.1)	103 (66.9)	
24–27.9	316 (7.6)	305 (7.6)	11 (7.2)	
≥28	81 (1.9)	78 (1.9)	3 (1.9)	
**Smoking during pregnancy (days/week)**				< 0.001
0	3,905 (93.3)	3,776 (93.7)	129 (83.8)	
1–2	179 (4.3)	165 (4.1)	14 (9.1)	
≥3	100 (2.4)	89 (2.2)	11 (7.1)	
**Alcohol drinking during pregnancy**				0.234
No	4,159 (99.4)	4,007 (99.4)	152 (98.7)	
Yes	25 (0.6)	23 (0.6)	2 (1.3)	
**Sleep duration during pregnancy (h/day)**				0.043
< 7	160 (3.8)	148 (3.7)	12 (7.8)	
7–8	1,403 (33.5)	1,356 (33.6)	47 (30.5)	
≥8	2,621 (62.7)	2,526 (62.7)	95 (61.7)	
**Morning sickness during pregnancy**				0.389
Yes	2,732 (65.3)	26 (65.2)	106 (68.8)	
No	1,452 (34.7)	1,404 (34.8)	48 (31.2)	
**Folic acid supplementation**				0.369
Yes	3,703 (88.5)	3,570 (88.6)	133 (86.4)	
No	481 (11.5)	460 (11.4)	21 (13.6)	
**Prenatal education during pregnancy (times/week)**				0.186
0	549 (13.1)	523 (13.0)	26 (16.9)	
1–2	1,858 (44.4)	1,786 (44.3)	72 (46.8)	
≥3	1,777 (42.5)	1,721 (42.7)	56 (36.3)	
**Diabetes mellitus before pregnancy**				0.370
Yes	81 (1.9)	80 (2.0)	1 (0.6)	
No	4,103 (98.1)	3,950 (98.0)	153 (99.4)	
**Gestational hyperglycemia including diabetes mellitus**				0.811
Yes	568 (13.6)	546 (13.5)	22 (14.3)	
No	3,616 (86.4)	3,484 (86.5)	132 (85.7)	
**Pregnancy-induced hypertension**				0.823
Yes	150 (3.6)	144 (3.6)	6 (3.9)	
No	4,034 (96.4)	3,886 (96.4)	148 (96.1)	
**Gestational anemia**				< 0.001
Yes	1,006 (24.0)	950 (23.6)	56 (36.4)	
No	3,178 (76.0)	3,080 (76.4)	98 (63.6)	
**Anxiety or depression during pregnancy**				0.121
Yes	109 (2.6)	102 (2.5)	7 (4.5)	
No	4,075 (97.4)	3,928 (97.5)	147 (95.5)	
**Postpartum anxiety or depression**				0.210
Yes	167 (4.0)	158 (3.9)	9 (5.8)	
No	4,017 (96.0)	3,872 (96.1)	145 (94.2)	
**Maternal exercise during pregnancy (min/day)**				< 0.001
< 20	924 (22.1)	866 (21.5)	58 (37.6)	
20–40	1,765 (42.2)	1,715 (42.5)	50 (32.5)	
≥40	1,495 (35.7)	1,449 (36.0)	46 (29.9)	

All variables are shown as n (%). *p*-values were calculated using chi-square test or Fisher's exact test.

ADHD, attention-deficit/hyperactivity disorder; BMI, body mass index.

Table 2 presents the binary logistic regression analyses of maternal exercise during pregnancy and ADHD among preschool children. Overall, compared with children whose mothers exercised for 20–40 min per day during pregnancy, children whose mothers exercised for < 20 min per day were more likely to be at risk of ADHD, with an OR of 2.11 (95% CI: 1.41–3.16, *p* < 0.001) after multivariable adjustment. However, the results were not statistically significant when comparing children whose mothers engaged in more than 40 min of exercise per day during pregnancy to those whose mothers exercised for 20–40 min per day. Logistic regression analyses suggested that boys had significantly higher odds of developing ADHD, with an odds ratio of 2.17 times; children whose mothers smoked during pregnancy had 2.47–3.44 times higher odds of developing ADHD; children whose mothers were diagnosed with gestational anemia had 1.77 times higher odds of developing ADHD after adjusting potential confounding factors (see Supplementary Table 1).

**Table 2:** OR (95% CI) for the association between maternal exercise during pregnancy and ADHD among preschool children.

	**ADHD/All cases**	**Crude model**	**Model 1^a^**	**Model 2^b^**	**Model 3^c^**
**Maternal exercise during pregnancy (min/day)**					
20–40	50/1,765	1.00 (Ref.)	1.00 (Ref.)	1.00 (Ref.)	1.00 (Ref.)
< 20	58/924	2.30 (1.56–3.38)	2.28 (1.55–3.36)	2.17 (1.45–3.23)	2.11 (1.41–3.16)
≥40	46/1,495	1.10 (0.73–1.64)	1.07 (0.72–1.61)	1.08 (0.71–1.63)	1.05 (0.69–1.58)

ADHD, attention-deficit/hyperactivity disorder; CI, confidence interval; OR, odds ratio; Ref., reference.

^a^Model 1 was adjusted for age and sex of the children.

^b^Model 2 was based on model 1, further adjusted maternal education level, BMI, smoking, alcohol drinking, prenatal education, sleep duration, morning sickness and folic acid supplementation during pregnancy.

^c^Model 3 was based on model 2, further adjusted diabetes mellitus before pregnancy, gestational hyperglycemia including diabetes mellitus, pregnancy-induced hypertension, gestational anemia, anxiety or depression during pregnancy, and postpartum anxiety or depression.

**Figure 1:**
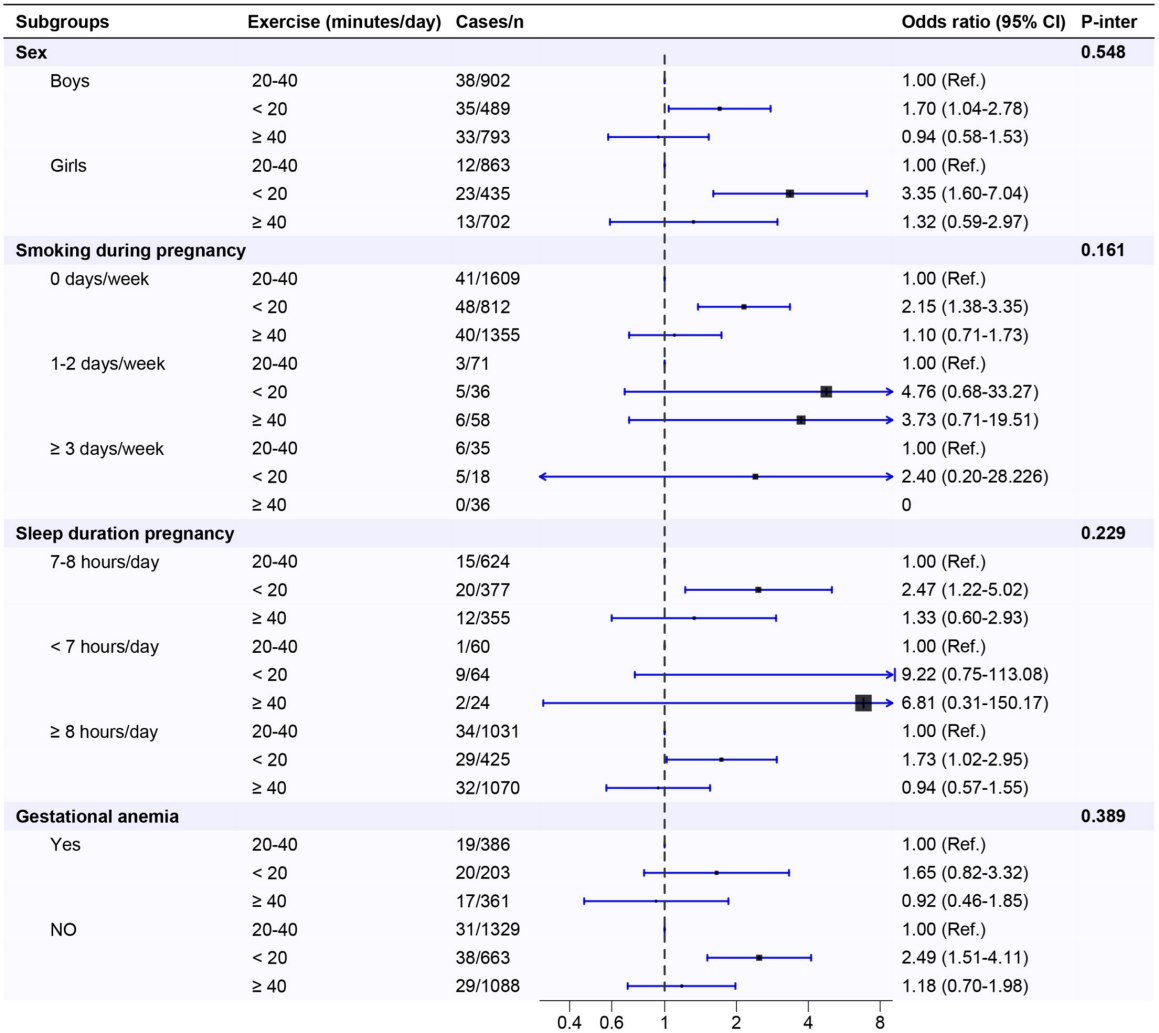
Maternal exercise during pregnancy in association with ADHD among preschool children by strata in the study population. ADHD, attention-deficit/hyperactivity disorder; BMI, body mass index.

The association between maternal exercise during pregnancy and childhood ADHD was similar in subgroups stratified by child's sex, as well as by maternal smoking, sleep duration and gestational anemia during pregnancy. No significant statistical interactions were observed between maternal exercise during pregnancy and these variables (all *p* for interaction > 0.05, Figure 1). Notably, the OR value for girls (OR = 3.35) whose mothers exercised < 20 min per day was approximately twice as high as that for boys (OR = 1.70), when compared to children whose mothers exercised 20–40 min per day during pregnancy.

## 4 Discussion

In this kindergarten-based cross-sectional study, we observed that preschool children in Southwest China whose mothers exercised for < 20 min per day during pregnancy, were more likely to exhibit symptoms of ADHD. The association between maternal exercise during pregnancy and childhood ADHD was independent of confounding factors, and was consistent across subgroups stratified by the child's sex, and by maternal smoking, sleep duration, and gestational anemia during pregnancy.

The World Health Organization (WHO) guidelines state that engaging in at least 150 min of moderate-intensity physical activity per week or 20–40 min per day during pregnancy is associated with numerous health benefits for both the mother and the baby (28). The present cross-sectional study found that 22.1% of the participants' mothers reported engaging in < 20 min of exercise during pregnancy. This finding suggested that low levels of exercise among Chinese pregnant women may have potential implications for offspring health, including an increased risk of metabolic disorders, impaired fetal growth, and negative effects on the child's neurodevelopment, as well as intelligence and language skills (29–32). Interestingly, our study revealed that engaging in < 20 min of exercise per day during pregnancy may be associated with an increased odds of childhood ADHD among preschool children in Southwest China, whereas moderate exercise of 20–40 min per day or more during pregnancy appears to provide prospective benefits against the development of childhood ADHD.

A growing body of evidence demonstrated that moderate exercise during pregnancy is safe and may be beneficial for early childhood neurodevelopment, including the child's speech and language, brain maturation, and cognitive development (33, 34). Our findings highlighted the importance of maternal moderate exercise during pregnancy for the prevention of childhood ADHD. So far, the exact biological mechanism linking maternal exercise during pregnancy and childhood ADHD remains unclear. The protective effect of moderate exercise during pregnancy on lowering the likelihood of childhood ADHD may be attributed to improved fetal neurodevelopment through the following possible mechanisms. Firstly, maternal exercise may increase placental functional capacity and enhance blood flow, facilitating the uptake and delivery of oxygen and nutrients to both the placenta and the developing fetus. This process is crucial for stimulating fetoplacental growth (35). Secondly, maternal exercise has been shown to potentially improve offspring memory by enhancing hippocampal neurogenesis (36). Regular exercise during pregnancy may have lasting positive effects on brain development and memory capabilities in children. Thirdly, maternal exercise may have a positive association with offspring neurodevelopment by regulating brain growth through mitochondrial metabolism (34). Improved mitochondrial function can increase energy availability for neuronal growth and the synthesis of neurotransmitters, ultimately supporting neurodevelopmental processes in offspring.

Previous studies on exploring risk factors for offspring ADHD have primarily focused on acquired factors of the children or maternal intrauterine exposure (37, 38). The present study primarily examined whether low levels of exercise during pregnancy are associated with an increased likelihood of ADHD in offspring. Our findings suggest that regular exercise during pregnancy may benefit fetal neurodevelopment, potentially reducing the likelihood of ADHD in offspring. A recent meta-analysis reported that physical exercise intervention could help alleviate the attention and executive function in children with ADHD (39). Additionally, our study found that boys had significantly higher odds of developing ADHD compared to girls. Moreover, maternal intrauterine exposures, such as smoking and gestational anemia during pregnancy, were associated with an increased likelihood of ADHD among preschool children. These findings are consistent with previous epidemiological studies reported in the literature (40, 41). Subgroup analyses further revealed that engaging in < 20 min of exercise per day during pregnancy may increase the likelihood of childhood ADHD across various stratification levels, indicating that this association between maternal exercise during pregnancy and ADHD among preschool children was consistent across different subgroups. No interaction effects were observed in the subgroup analyses. The absence of interaction effects implies that the association between maternal exercise during pregnancy and the likelihood of childhood ADHD does not significantly vary based on these subgroup stratified variables. Notably, subgroup analyses revealed that engaging in < 20 min of exercise per day during pregnancy may have a greater detrimental impact on the likelihood of developing ADHD in girls than in boys. The potential of maternal exercise during pregnancy differentially affects the development of ADHD in girls vs. boys presents a promising avenue for future research in this domain. Consequently, future prospective studies are needed to confirm the potential benefits of enhancing maternal exercise levels during pregnancy in reducing the likelihood of childhood ADHD among preschool children, with a particular emphasis on research involving different genders.

To our knowledge, our study is the first to examine the association between maternal exercise during pregnancy and ADHD among Chinese preschool children. We also considered a wide range of potential confounders to better estimate the association between maternal exercise during pregnancy and childhood ADHD. Otherwise, we conducted sufficient sampling at the survey site to ensure a high regional sample representation of the Southwest China. However, it is important to mention several limitations of the present study to encourage caution when interpreting the findings. First, due to the cross-sectional design, it is difficult to establish definitive causal relationships between maternal exercise during pregnancy and childhood ADHD. Second, the revised CPSQ was used to assess childhood ADHD rather than clinical diagnosis, thus reporting bias may exist. And recall bias is attributed to the self-report by the children's parents, which may have biased the observed risk association. Finally, some unknown or unmeasured variables were not assessed in this study, such as maternal exposure to teratogens, drugs and other pollutants, which may be associated with childhood ADHD.

The preschool period represents a crucial phase in a child's physical and mental development (42). During this period, ADHD is one of the most prevalent neurodevelopmental disorders (43). Various factors during pregnancy can influence fetal brain development, which may predispose children to ADHD later in life (44). The current findings showed that less maternal exercise during pregnancy was associated with an increased likelihood of ADHD among preschool children in Southwest China. It may suggest a potential role for maternal exercise during pregnancy in preventing childhood ADHD. Therefore, we strongly recommend that prenatal health education emphasize the significance of moderate-intensity maternal exercise during pregnancy to promote the long-term physical and mental health of children. However, prospective studies are needed to elucidate the causality between maternal exercise during pregnancy and the risk of childhood ADHD.

## Data Availability

The raw data supporting the conclusions of this article will be made available by the authors, without undue reservation.
