# Supplementary material for: Association between maternal exercise during pregnancy and attention-deficit/hyperactivity disorder among preschool children in Southwest China
Source: Front Public Health. 2024 Nov 27;12:1493580. doi: 10.3389/fpubh.2024.1493580 (PMC11631731; doi:10.3389/fpubh.2024.1493580)
Supplement: Supplementary file 1 [file Table_1.docx]

Supplementary Material

SUPPLEMENTARY TABLE 1. Associations of children’s sex, maternal smoking, sleep duration, and gestational anemia with ADHD among preschool children.

| **Characteristic** | **Unadjusted**  **odds ratio ^a^ (95% CI)** | **Adjusted**  **odds ratio ^b^ (95% CI)** |
| --- | --- | --- |
| Sex |  |  |
| Girls | 1.00 (Ref.) | 1.00 (Ref.) |
| Boys | 2.08 (1.47-2.94) | 2.17 (1.52-3.13) |
| Smoking during pregnancy (days/week) |  |  |
| 0 | 1.00 (Ref.） | 1.00 (Ref.） |
| 1-2 | 2.48 (1.40-4.41) | 2.47 (1.37-4.44) |
| ≥ 3 | 3.62 (1.89-6.93) | 3.44 (1.75-6.77) |
| Sleep duration during pregnancy (hours/day) |  |  |
| 7-8 | 1.00 (Ref.) | 1.00 (Ref.） |
| < 7 | 2.34 (1.21-4.51) | 1.94 (0.97-3.88) |
| ≥ 8 | 1.09 (0.76-1.55) | 1.07 (0.74-1.53) |
| Gestational anemia |  |  |
| No | 1.00 (Ref.) | 1.00 (Ref.） |
| Yes | 1.85 (1.32-2.59) | 1.77 (1.24-2.51) |

^a^ Odds ratio was calculated using binary logistic regression.

^b^ Adjusted for all covariates except itself.

ADHD = attention-deficit/hyperactivity disorder; CI = confidence interval; Ref. = Reference.
